# Development of a simultaneous LC–MS/MS analytical method for plasma: 16 antipsychotics approved in Japan and 4 drug metabolites

**DOI:** 10.1007/s44211-024-00619-2

**Published:** 2024-06-25

**Authors:** Masamitsu Maekawa, Maki Yokota, Toshihiro Sato, Yu Sato, Masaki Kumondai, Yuji Sato, Masato Suzuka, Daisuke Kobayashi, Kotaro Sakamoto, Masaki Matsuura, Masafumi Kikuchi, Hiroshi Komatsu, Kumiko Fujii, Yuji Ozeki, Hiroaki Tomita, Nariyasu Mano

**Affiliations:** 1 Faculty of Pharmaceutical Sciences, Tohoku University, 1-1 Seiryo-machi, Aoba-ku, Sendai, 980-8574 Japan; 2 Department of Pharmaceutical Sciences, Tohoku University Hospital, 1-1 Seiryo-machi, Aoba-ku, Sendai, 980-8574 Japan; 3 Department of Psychiatry, Tohoku University Graduate School of Medicine, Sendai, 980-8574 Japan; 4 Department of Psychiatry, Shiga University of Medical Science, Otsu, 520-2192 Japan

**Keywords:** Antipsychotics, LC–MS/MS, Schizophrenia, Simultaneous analysis, Plasma drug concentrations

## Abstract

The increased risk of adverse drug reactions due to the concomitant use of antipsychotics is problematic in the treatment of schizophrenia. Therefore, the simultaneous analysis of their plasma concentrations is required. In this study, we developed a simultaneous liquid chromatography/tandem mass spectrometry (LC–MS/MS) method for analyzing plasma antipsychotics approved in Japan for therapeutic drug monitoring (TDM) applications. First, we counted the prescriptions for 16 antipsychotics and concomitant drugs used at the Tohoku University Hospital. LC–MS/MS was used for the simultaneous analysis of 16 antipsychotics and four drug metabolites. This analysis was conducted using a combination of selected reaction monitoring mode and reversed-phase chromatography. Following the examination of the MS/MS and LC conditions, an analytical method validation test was conducted. The developed method was used to analyze plasma antipsychotic levels in patients with schizophrenia. One-third of the patients received treatment with multiple antipsychotics. Under LC–MS/MS conditions, LC separation was performed using a combination of a C18 column and ammonium formate-based mobile phases with a gradient flow. The calibration curves were optimized by adjusting the ion abundance, and 11 compounds met the criteria for intra- and inter-day reproducibility tests. Some stability test results did not meet these criteria; therefore, further investigation is required. The developed method permitted the measurement of all the plasma parameters, including concentrations above the therapeutic range. Therefore, this method may be useful in the daily TDM practice of antipsychotics.

**Graphical abstract:**

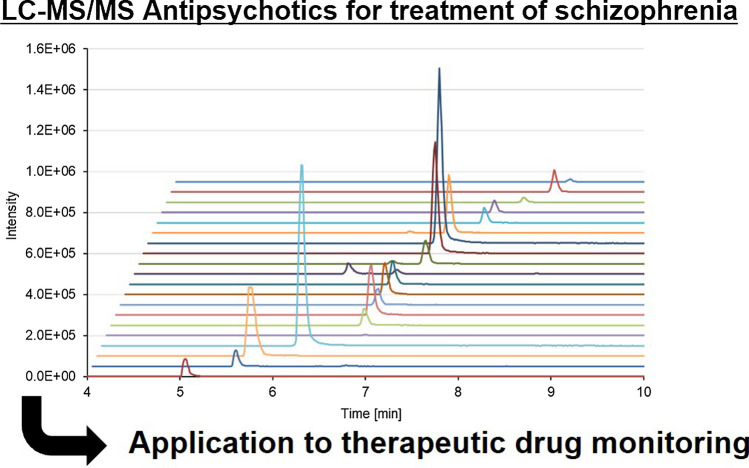

**Supplementary Information:**

The online version contains supplementary material available at 10.1007/s44211-024-00619-2.

## Introduction

Schizophrenia is a common mental disorder with a lifetime prevalence of approximately 0.5% [1, 2], and it is estimated that approximately 800,000 individuals in Japan are affected by the disease. Symptoms of this condition include positive (delusions, hallucinations, and auditory hallucinations), negative (hyperesthesia, anhedonia, decreased motivation, apathy, and aphasia), and cognitive dysfunction (decreased attention, concentration, and memory) [3, 4]. Treatment was administered using a combination of pharmacotherapy and psychosocial treatments [5]. Antipsychotics can be classified into two categories: typical and atypical. Typical antipsychotics, including chlorpromazine and haloperidol, primarily act on dopamine D2 receptors, resulting in improvements in the positive symptoms [4, 6]. Atypical antipsychotics also inhibit receptors other than D2. For example, risperidone, which is a serotonin/dopamine antagonist, inhibits serotonin 5-HT2A receptors, while clozapine, which is classified as a multi-receptor antipsychotic, inhibits muscarinic M1, adrenergic α1, and histamine H1 receptors in addition to dopamine and serotonin receptors [6, 7]. Aripiprazole, a partial dopamine agonist, is also included in the atypical antipsychotics. Atypical antipsychotics have been shown to be effective in the treatment of both positive and negative symptoms, as well as cognitive dysfunction. The prevalence of extrapyramidal side effects was significantly lower with these medications than with typical antipsychotics [8]. It is crucial to administer drug therapy to prevent the recurrence of schizophrenia and to maintain the quality of life (QOL) of affected individuals [5].

Antipsychotic monotherapy is generally recommended for the pharmacological treatment of schizophrenia [6]. In cases where multiple therapeutic approaches, including clozapine treatment, have proven ineffective [9, 10], Japanese guidelines recommend the consideration of combined antipsychotics [5]. Nevertheless, the concomitant use of antipsychotics is a prevalent phenomenon. A Japanese survey revealed that 55% of patients in Japan were receiving combination antipsychotics [11]. However, the efficacy and safety of multiple antipsychotics remain unclear. With regard to safety, there are concerns regarding the continuation of drug therapy, given the increasing number of anticholinergic drugs employed to prevent and treat extrapyramidal symptoms [12–14]. These drugs are associated with an increased risk of death [15, 16], metabolic abnormalities [17], and QT prolongation [18]. A significant proportion of antipsychotics are metabolized by cytochrome P450 (CYP) and UDP-glucuronosyltransferases. The concomitant use of antipsychotics may alter their pharmacokinetics owing to drug–drug interactions (DDIs) [19]. Additionally, reports have indicated that alterations in pharmacokinetics may result in the emergence of adverse effects and exacerbation of preexisting symptoms. Therefore, caution should be exercised when considering DDIs [20–25].

Guidelines for the therapeutic drug monitoring (TDM) of psychotropics have been established in other countries [19]. Conversely, in Japan, TDM is not used for antipsychotics other than haloperidol and bromperidol. The efficacy of TDM for antipsychotics has not yet been established in Japan, given the differences in approved drugs between Japan and other countries. Consequently, it is imperative to ascertain the efficacy of TDM for the antipsychotic drugs approved in Japan.

Analytical methods for antipsychotics include high-performance liquid chromatography (HPLC)/ultraviolet detection [26–28], HPLC/fluorescence detection [29], HPLC/mass spectrometry (MS), and GC–MS [30]. In recent years, numerous methods employing liquid chromatography–tandem mass spectrometry (LC–MS/MS), which offer high sensitivity and specificity, have been reported [31–37]. The utility of simultaneous analysis methods for routine TDM has also been reported [37–40]. However, a simultaneous analysis method that focuses on antipsychotics approved in Japan is yet to be reported.

The objective of this study was to assess the utility of therapeutic drug monitoring (TDM) using LC–MS/MS for antipsychotics approved in Japan. First, a survey was conducted at the Tohoku University Hospital to investigate the prescription trends of antipsychotics in conjunction with the potential for DDIs via CYP. Subsequently, we developed a method for the simultaneous analysis of 16 antipsychotics and four active drug metabolites. Four drug metabolites were included in this study: dehydroaripiprazole, *N*-desmethylclozapine, clozapine-*N*-oxide, and *N*-desalkylquetiapine. Dehydroaripiprazole is an active metabolite of aripiprazole, formed by both CYP2D6 and CYP3A4 [41]. Clozapine is metabolized into two major active metabolites, *N*-desmethylclozapine and clozapine-*N*-oxide, by CYP1A2 and/or CYP3A4 [42]. *N*-Desalkylquetiapine is an active metabolite of CYP2D6 [43]. Active metabolites are useful for pharmacodynamics and pharmacokinetics as well as for calculating the ratio of drugs to metabolites [19, 43]. Following the optimization of MS/MS conditions and investigation of LC conditions, the reliability of this method was confirmed using an analytical method validation test. Finally, this method was employed to assess plasma antipsychotic concentrations in patients with schizophrenia.

## Experimental

### Chemicals

Ammonium formate, aripiprazole, formic acid, perospirone hydrochloride dihydrate, and quetiapine fumarate were purchased from FUJIFILM Wako Pure Chemical Corporation (Osaka, Japan). Dehydroaripiprazole, blonanserin-d_5_, and olanzapine-d_3_ were purchased from Toronto Research Chemicals (North York, NY, Canada). Asenapine maleate, *N*-desmethylclozapine, and *N*-desalkylquetiapine were purchased from Sigma-Aldrich (St. Louis, MO, USA). Blonanserin, chlorpromazine hydrochloride, levomepromazine maleate, olanzapine, paliperidone, perphenazine, risperidone, and sulpiride were purchased from Tokyo Kasei Kogyo Co., Ltd. (Tokyo, Japan). Brexpiprazole, zotepine, and clozapine-*N*-oxide were purchased from Cayman Chemical Company (Ann Arbor, MI, USA). Clozapine and quetiapine-d_8_ fumarate were purchased from Santa Cruz Biotechnology (Dallas, TX, USA). Lurasidone was purchased from Ado-OQ BioScience (Irvine, CA, USA). Clozapine-d_8_ was purchased from R&D Systems Inc. (Minneapolis, MN, USA). Amitriptyline-d_3_, clomipramine-d_3_, and desipramine-d_3_ were purchased from Cambridge Isotope Laboratories (Tewksbury, MA, USA). Acetonitrile was purchased from Kanto Kagaku Co. Ltd. (Tokyo, Japan). Methanol was purchased from FUJIFILM Wako Pure Chemical Corporation and used after distillation and filtration. Ethanol was purchased from Nacalai Tesque, Inc. (Kyoto, Japan). Heparinized human plasma was supplied by Cosmo Bio Co., Ltd. (Tokyo, Japan). Ultrapure water was prepared using a Puric-α (Organo Co., Ltd., Tokyo, Japan).

### LC/MS/MS equipment

A Nexera high-performance liquid chromatograph (Shimadzu, Kyoto, Japan) was connected to an API 5000 triple quadrupole mass spectrometer (SCIEX, Framingham, MA, USA) equipped with an ESI probe. All data were analyzed using the Analyst 1.5.0 software (SCIEX).

### Preparation of standard and internal standard solutions

After weighing the analytes, the stock solutions of dehydroaripiprazole (315 μg/mL), asenapine (402 μg/mL), blonanserin (1.39 mg/mL), brexpiprazole (562 μg/mL), chlorpromazine (1.40 mg/mL), clozapine (1.28 mg/mL), *N*-desmethylclozapine (353 μg/mL), clozapine-*N*-oxide (210 μg/mL), levomepromazine (597 μg/mL), lurasidone (552 μg/mL), olanzapine (525 μg/mL), perospirone (125 μg/mL), perphenazine (758 μg/mL), quetiapine (1.06 mg/mL), *N*-desalkylquetiapine (581 μg/mL), risperidone (1.16 mg/mL), and zotepine (556 μg/mL) were prepared by dissolution with ethanol. The solutions of aripiprazole (1.03 mg/mL) and sulpiride (1.29 mg/mL) were prepared by mixing ethanol with 5% acetic acid. A solution of paliperidone (1.48 mg/mL) was prepared using a mixture of ethanol and ethyl acetate (1:1, v/v). The solutions were mixed and diluted to prepare standard solutions for calibration and quality control (QC). The concentrations of the calibration curves and QC samples are summarized in Table S1. For internal standard (IS), after preparing a solution of 100 μg/mL for blonanserin-d_5_, clozapine-d_8_, olanzapine-d_3_, and quetiapine-d_8_ and 10 μg/mL for amitriptyline-d_3_, clomipramine-d_3_, and desipramine-d_3_, the solutions were diluted and mixed to prepare a 10 ng/mL IS working solution. All prepared solutions were stored at -20°C in glass vials.

### Prescription trend survey

Sixteen antipsychotics were prescribed, namely, aripiprazole, asenapine, blonanserin, brexpiprazole, chlorpromazine, clozapine, levomeprimazine, lurasidone, olanzapine, paliperidone, perospirone, perphenazine, quetiapine, risperidone, sulpiride, and zotepine. The number of prescriptions was counted using the drug prescription management software Yunicom-GX (Yuyama MFG Co., Ltd., Osaka, Japan). Prescription analyses were conducted using Microsoft Access 2019 and Microsoft Excel 2019. We extracted prescriptions for antipsychotics from the psychiatric department between January 1 and December 31, 2020. The number of antipsychotic drugs administered was determined. Subsequently, the maximum duration of use was determined by employing the following “formula 1,” derived from the prescription date and the number of days' supply of antipsychotics.

1 $${\text{Maximum day of use}}\,{ = }\,{\text{prescription date}}\,{ + }\,{\text{prescription days}}$$

Drugs prescribed between the date of the initial prescription and the date of the maximum prescription were considered concomitant drugs. Prescriptions of concomitant drugs were enumerated from all clinical departments. Seventeen antipsychotics, including haloperidol and its concomitant drugs, were selected for the analysis. The number of prescriptions for concomitant drug use was counted. A review was conducted on the possibility of DDIs between metabolic enzymes, CYP-inhibiting drugs, and CYP-inducing drugs, based on Japanese package inserts, Japanese interview forms, and scientific papers. Subsequently, the potential combinations of DDIs were identified.

### LC/MS/MS conditions

MS/MS was performed in positive ion mode. The following parameters were set for the mass spectrometer: curtain gas (CUR), collision gas (CAD), interspray voltage (ISV), turbo gas temperature (TEM), nebulizer gas (GS1), and turbo heater gas (GS2). These were set to 15, 10, 5500 V, 700 °C, 40, and 60 psi, respectively. For infusion MS/MS optimization, each standard solution was diluted to a concentration of 1–100 ng/mL and applied at a flow rate of 10 μL/min. The SRM conditions, including the precursor ions (*m/z*), product ions (*m/z*), collision energy (CE), cell exit potential (CXP), declustering potential (DP), and entrance potential (EP), were optimized. Subsequently, the analytes were diluted to 2.5 ng/mL, and ionization parameters were optimized using flow injection analysis (FIA). Mobile phases A and B (1:4, v/v) were injected at a rate of 0.3 mL/min for FIA optimization. The following variables were investigated ranging from 15 to 25 psi, 3 to 12 psi, 40 to 80 psi, 40 to 80 psi, 4000 to 5000 V, and 300 to 700 °C, respectively: CUR, CAD, GS1, GS2, ISV, and TEM. Additionally, we conducted research on DP, EP, and CXP within the specified voltage ranges of 10–90, 2–12, and 10–50 V, respectively. Mixtures of 10 mmol/L ammonium formate in water/formic acid (100:0.1, v/v) and 10 mmol/L ammonium formate in methanol/formic acid (100:0.1, v/v) were used as mobile phases A and B, respectively. The flow rate was set at 0.3 mL/min. LC separation was performed in gradient mode as follows: mobile phase B (%), 5 > 5 > 60 > 100 > 100, time (min), 0 > 3 > 4 > 8 > 10. The equilibration time was set to 3 min. The analytical column employed was the YMC-Triart C18 (2.1 mm i.d. × 150 mm, 3 μm, YMC, Kyoto), with a column temperature of 40 °C. A 15 μL sample solution was injected.

### Calibration curves

Standard solutions for the calibration curves were prepared by diluting mixed standard solutions with ethanol. The concentrations of the calibration curves are summarized in Table S1a. To 20 μL of blank plasma, 20 μL of the standard for calibration (or only solvent, ethanol), 20 μL of internal standard, and 40 μL of acetonitrile were added and mixed. The samples were centrifuged at 15,000×*g* for 10 min at 4 °C, and 70 μL of the supernatant was transferred to another tube. After evaporation in a centrifugal evaporator at 40°C, the samples were redissolved in 70 μL of 40% methanol, and 15 μL was subjected to LC–MS/MS analysis.

Calibration curves were constructed by plotting the concentrations of the analytes against the peak area ratio to IS and performing linear or quadratic regression using the least-squares method with 1/*x*^2^ weighting. In the instances of ion saturation, the CE and/or DP values were adjusted to prevent ion suppression.

### Analytical method validation

The analytical method was validated in accordance with the guidelines provided by the US Food and Drug Administration [44]. To assess the selectivity of the method, 20 μL of blank plasma, 40 μL of ethanol, and 40 μL of acetonitrile were added to the sample and mixed. Subsequent procedures were identical to those described previously. The selectivity of the method was validated by demonstrating that endogenous substances did not interfere with the peak of the target compound. The criteria were as follows: in both standard and IS-free blank plasma samples, no analyte peaks and less than 5% of the IS peak area were detected.

To evaluate the matrix effect, 20 μL of the QC standard solution (MQC), 20 μL of the internal standard solution, and 40 μL of acetonitrile were added to 20 μL of blank plasma or water, and the mixture was then vortexed. Subsequent procedures were identical to those described previously. Blank plasma and water were analyzed three times, and the matrix factor (MF) and IS-normalized MF were calculated using the following formulae Eqs. (2) and (3) to evaluate the matrix effect.

2 $${\text{MF }}\left( {\text{\% }} \right)\,{ = }\,{\text{PA}}_{{\text{P}}} {\text{/PA}}_{{\text{S}}} \, \times \,{100}$$

PA_P_ is the Peak area of the analytes or ISs in the plasma sample.

PA_S_ is the Peak area in the standard solution (without sample matrix).

3 $${\text{IS normalized MF }}\left( {\text{\% }} \right)\,{ = }\,{\text{MF}}_{{\text{A}}} {\text{/MF}}_{{\text{I}}} \, \times \,{100}$$

MF_A_ is the MF of the analytes.

MF_I_ is the MF of ISs.

To assess carryover, 20 μL of the standard solution, 20 μL of the IS solution, and 40 μL of acetonitrile were combined with 20 μL of blank plasma to prepare a standard sample. Additionally, a blank sample was prepared by adding 40 μL of ethanol and 40 μL of acetonitrile to 20 μL of blank plasma and mixing. Following the measurement of the highest-concentration standard solution, a blank sample was measured sequentially. Carryover was evaluated by calculating the ratio of the peak area of the analytes in the blank to that of the analytes in the highest standard solution. A wash run was conducted between standard and blank measurements. In the wash run, the concentration of mobile phase B was set to 100%, and 15 μL of methanol was injected for 0.5 min, which was repeated five times. Equilibration analysis was conducted between the wash run and the blank. In this analysis, the concentration of mobile phase B was set to 5%, 1 μL of methanol was injected, and the measurement was performed for 5 min.

For the intra- and inter-day reproducibility testing, 20 μL of the QC standard solution, 20 μL of the IS solution, and 40 μL of acetonitrile were added to 20 μL of blank plasma, mixed, and analyzed in accordance with the previously described methodology. The lower limit of quantification (LLOQ) and three concentrations (LQC, MQC, and HQC) of QC samples were measured. In this study, each concentration was analyzed six times, and in the daily variation study, each concentration was analyzed over three days. Data accuracy and precision were evaluated using the relative error (RE) and coefficient of variation (CV), respectively. The QC concentration and formulae for calculating the RE and CV (Eqs. (4) and (5) are presented below.

4 $${\text{RE }}\left[ \% \right]{\mkern 1mu} { = }{\mkern 1mu} \left( {{\text{average value of quantitative values}} - {\text{theoretical value}}} \right){\text{ / theoretical value}}{\mkern 1mu} \times {\mkern 1mu} {100}$$

5 $${\text{CV }}\left[ {\text{\% }} \right]\,{ = }\,{\text{standard deviation of quantitative values/average value of quantitative values}}\, \times {100}$$

For stability testing, 20 μL of the QC standard solution was evaporated to dryness using a centrifugal evaporator (at 40°C for 1 h), and 20 μL of blank plasma was subsequently added. After storage at -80°C for 1, 7, 14, and 28 days, or at 4°C for 2, 6, and 24 h, or at 25°C for 2 and 24 h, the samples were analyzed as previously described. Each concentration was analyzed in triplicate using the LQC and HQC samples described above. The storage stability was assessed using both RE and CV.

### Measurement of plasma samples of the patients with schizophrenia

This study was conducted as a multicenter collaborative study approved by the Ethical Review Committee of Tohoku University Graduate School of Medicine and Shiga University of Medical Science Graduate School of Medicine (approval number: 2019-1-525). After the addition and mixing of 20 μL of ethanol, 20 μL of internal standard solution, and 40 μL of acetonitrile to 20 μL of patient plasma, the same procedure as previously described above was performed, and the mixture was subjected to analysis. Quantification was performed using a calibration curve measured using the same analytical lot.

## Result and discussion

### Prescription of antipsychotic drugs

First, we counted the number of prescriptions of antipsychotic drugs at the Department of Psychiatrics, Tohoku University Hospital. Antipsychotics are also used to treat non-psychiatric conditions such as delirium, nausea, and vomiting during cancer treatment [45–47]. To exclude their use for these purposes, the count was limited to prescriptions from psychiatric departments. The total number of prescriptions for the 16 antipsychotics was 1,751 in 1 year, with aripiprazole being the most frequently prescribed drug, with 325 prescriptions (Table S2a). The survey revealed that 3,738 concomitant drug combinations with antipsychotics were prescribed. Of these, 74 combinations with potential CYP-mediated DDIs were prescribed 430 times (Table S2b). The results indicated that levomepromazine was the most frequently prescribed medication (103 times), followed by sertraline and fluvoxamine (Table S2b). Antipsychotics have been reported to alter pharmacokinetics through CYP-mediated DDIs, affecting their efficacy and safety [21–24, 48]. The results of this study highlight the significance of plasma concentration analysis in ensuring the safe and effective use of antipsychotic drugs. Our findings indicate a clear need for the development of a method for simultaneous analysis of antipsychotic drugs.

### Investigation of analytical conditions for the development of a simultaneous LC/MS/MS analysis method

Following the prescription survey, we developed a method for the simultaneous analysis of antipsychotics using LC–MS/MS. The analytes included 16 antipsychotics approved in Japan and four active metabolites. In comparison to previous studies, the analytes in this study were not fully covered in any previous paper [38–40, 49, 50]; thus, the novelty of this study lies in its simultaneous analysis of Japanese-approved antipsychotics.

First, SRM conditions exhibiting the highest intensity for the analytes and ISs were examined. In the MS spectra, protonated molecules ([M + H]^+^) were detected as base peaks in positive ion mode for all compounds. The product ions and DP, CE, EP, and CXP values were obtained by maximizing the intensities. Table 1a presents the SRM parameters for detection at maximum intensities. Subsequently, the ionization conditions were optimized. Consequently, it was determined that there was minimal variation in the ionization behavior of each compound. The values for CUR, CAD, ISV, TEM, GS1, and GS2 were set to 20 psi, 12 psi, 5000 V, 700 ºC, 50 psi, and 70 psi, respectively.

**Table 1:** SRM parameters of all compounds.

Compounds	Category	Q1 (*m/z*)	Q3 (*m/z*)	DP (V)	EP (V)	CE (V)	CXP (V)
(a) SRM parameters for detection at the most intense							
Aripiprazole	Analyte	448.3	287.2	50	2	37	22
Dehydroaripiprazole	Analyte	446.2	285.2	30	4	33	26
Asenapine	Analyte	286.2	44.0	80	2	63	10
Blonanserin	Analyte	368.4	297.3	30	2	53	26
Brexpiprazole	Analyte	434.2	273.3	60	2	35	28
Chlorpromazine	Analyte	319.3	86.2	40	2	25	18
Clozapine	Analyte	327.2	84.1	30	2	31	12
* N*-Desmethylclozapine	Analyte	313.2	270.1	90	2	25	26
Clozapine-* N*-oxide	Analyte	343.3	192.1	50	2	59	14
Levomepromazine	Analyte	329.3	58.0	40	2	61	12
Lurasidone	Analyte	493.4	166.2	20	2	57	16
Olanzapine	Analyte	313.2	256.1	40	2	31	24
Paliperidone	Analyte	427.3	207.4	150	2	41	14
Perospirone	Analyte	427.3	177.3	20	2	49	16
Perphenazine	Analyte	404.3	171.1	10	2	33	18
Quetiapine	Analyte	384.3	253.2	10	2	31	20
* N*-Desalkylquetiapine	Analyte	296.3	210.2	70	2	41	14
Risperidone	Analyte	411.3	191.1	20	2	41	16
Sulpiride	Analyte	342.3	112.3	50	2	35	16
Zotepine	Analyte	332.2	71.9	30	2	29	10
Blonanserin-d_5_	IS	373.4	297.1	40	4	37	16
Clozapine-d_8_	IS	335.3	275.3	50	2	31	20
Olanzapine-d_3_	IS	316.3	256.2	50	2	33	20
Quetiapine-d_8_	IS	392.3	257.9	91	2	37	32
Amitriptyline-d_3_	IS	281.3	91.0	30	2	31	14
Clomipramine-d_3_	IS	318.4	89.1	60	2	27	12
Desipramine-d_3_	IS	270.4	75.4	90	2	23	14
(b) SRM parameters for prevention saturation of calibration curves with ion adjustment techniques							
Aripiprazole	Analyte	448.3	287.2	50	2	37	22
Dehydroaripiprazole	Analyte	446.2	285.2	30	4	33	26
Asenapine	Analyte	286.2	44.0	80	2	63	10
Blonanserin	Analyte	368.4	297.3	30	2	53	26
Brexpiprazole	Analyte	434.2	273.3	60	2	35	28
Chlorpromazine	Analyte	319.3	86.2	40	2	25	18
Clozapine	Analyte	327.2	84.1	30	2	31	12
* N*-Desmethylclozapine	Analyte	313.2	270.1	90	2	25	26
Clozapine-*N*-oxide	Analyte	343.3	192.1	50	2	59	14
Levomepromazine	Analyte	329.3	58.0	40	2	61	12
Lurasidone	Analyte	493.4	166.2	20	2	57	16
Olanzapine	Analyte	313.2	256.1	40	2	31	24
Paliperidone	Analyte	427.3	207.4	150	2	41	14
Perospirone	Analyte	427.3	177.3	20	2	49	16
Perphenazine	Analyte	404.3	171.1	10	2	33	18
Quetiapine	Analyte	384.3	253.2	10	2	31	20
* N*-Desalkylquetiapine	Analyte	296.3	210.2	70	2	41	14
Risperidone	Analyte	411.3	191.1	20	2	41	16
Sulpiride	Analyte	342.3	112.3	50	2	35	16
Zotepine	Analyte	332.2	71.9	30	2	29	10
Blonanserin-d_5_	IS	373.4	297.1	40	4	37	16
Clozapine-d_8_	IS	335.3	275.3	50	2	31	20
Olanzapine-d_3_	IS	316.3	256.2	50	2	33	20
Quetiapine-d_8_	IS	392.3	257.9	91	2	37	32
Amitriptyline-d_3_	IS	281.3	91.0	30	2	31	14
Clomipramine-d_3_	IS	318.4	89.1	60	2	27	12
Desipramine-d_3_	IS	270.4	75.4	90	2	23	14

*CE* Collision energy, *CED* collision energy defect, *CXP* cell exit potential, *DP* declustering potential, *EP* entrance potential, *IS* internal standard

**Fig. 1:**
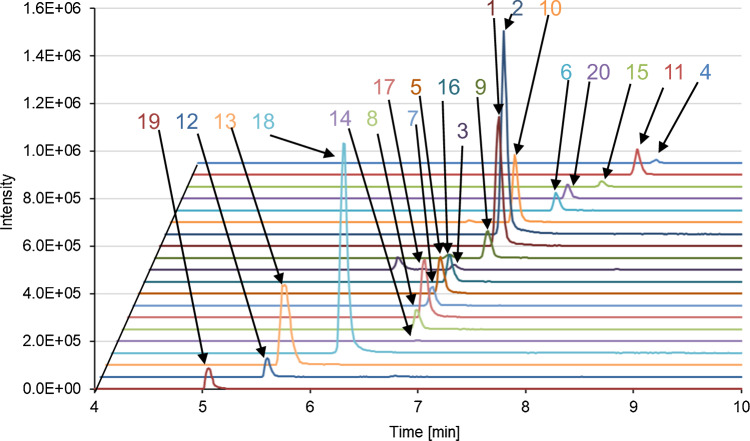
SRM chromatograms of the analytes. 1 aripiprazole, 2 dehydroaripiprazole, 3 asenapine, 4 blonanserin, 5 brexpiprazole; 6 chlorpromazine, 7 clozapine, 8 *N*-desmethylclozapine, 9 clozapine-*N*-oxide, 10 levomepromazine, 11 lurasidone, 12 olanzapine, 13 paliperidone, 14 perospirone, 15 perphenazine, 16 quetiapine, 17 *N*-desalkylquetiapine, 18 risperidone, 19 sulpiride, 20 zotepine

Some pretreatment methods, including solid-phase extraction [31, 32] and liquid–liquid extraction [33], have been employed to remove plasma interference in antipsychotic LC–MS/MS. However, these methods have the disadvantage of being time-consuming in comparison to protein precipitation [32, 35, 40, 50]. To align with the prevailing practices of daily TDM, we employed a straightforward protein precipitation method for plasma pretreatment. Therefore, we decided to perform the LC separation of these drugs. The YMC-Triart C18 column (2.1 mm i.d. × 150 mm, 3 μm) was employed as the analytical column, and gradient conditions were investigated. As a result of the optimization, the proportion of mobile phase B was increased to 5% from 0 to 3 min, 5% to 60% from 3 to 4 min, 60% to 100% from 4 to 8 min, and 100% from 8 to 10 min. Under these conditions, the analytes were fully separated (Fig. 1). Calibration curves were prepared using the aforementioned LC–MS/MS conditions. First, asenapine, brexpiprazole, chlorpromazine, clozapine, *N*-desmethylclozapine, clozapine-*N*-oxide, levomepromazine, lurasidone, olanzapine, perospirone, quetiapine, *N*-desalkylquetiapine, sulpiride, and zotepine were saturated at high concentrations (data not shown). Consequently, various ion abundance methods have been employed to adjust the aforementioned methods. The following methods are known to adjust the ion amounts [51, 52]: collision energy defect (CED) [53], in-source CID (is-CID) [54–57], by-product ion SRM [58], and isotopologue SRM [59]. In this study, CED and is-CID were applied, and the SRM conditions were changed (Table 2). CED was successful in the case of asenapine, brexpiprazole, chlorpromazine, *N*-desmethylclozapine, lurasidone, olanzapine, paliperidone, perospirone, *N*-desalkylquetiapine, sulpiride, and zotepine, respectively, and the is-CID technique was useful in the case of clozapine,* N*-desmethylclozapine, clozapine-*N*-oxide, levomepromazine, and sulpiride, respectively. For clozapine, *N*-desmethylclozapine, and sulpiride, both CED and is-CID were used for adjustment. The use of CED and is-CID resolved the saturation of the calibration curve and expanded the calibration range beyond that achievable without ion abundance reduction (Table 2a).

**Table 2:** The result of analytical method validation items

Analytes	Range (ng/mL)	Equation	*R*^2^
(a) Calibration curves of analytes			
Aripiprazole	5 – 1500	y = 0.00376 x + 0.0044	0.9984
Dehydroaripiprazole	1 – 300	y = 0.0271 x + 0.00579	0.9986
Asenapine	0.24 – 12	y = 0.0264 x + 0.0315	0.9969
Blonanserin	0.03 – 3	y = 0.0485 x + 0.000347	0.9965
Brexpiprazole	2 – 300	y = 0.0118 x—0.000606	0.9990
Chlorpromazine	5 – 750	y = 0.000544 x + 0.000627	0.9972
Clozapine	10 – 3000	y = 0.000317 x + 0.000938	0.9978
* N*-Desmethylclozapine	10 – 1500	y = 0.000356 x + 0.000183	0.9982
Clozapine-*N*-oxide	10 – 1500	y = 0.000416 x + 0.00123	0.9977
Levomepromazine	3 – 900	y = 0.0085 x + 0.00342	0.9973
Lurasidone	0.6 – 180	y = 0.00792 x + 0.000161	0.9966
Olanzapine	1.4 – 420	y = 0.00166 x + 0.000203	0.9983
Paliperidone	0.6 – 180	y = 0.0268 x + 0.000785	0.9985
Perospirone	0.3 – 15	y = 0.00129 x + 0.000116	0.9964
Perphenazine	0.12 – 12	y = 0.00231 x^2 + 0.0851 x—0.00238	0.9981
Quetiapine	8 – 2400	y = 0.00212 x—0.00133	0.9983
* N*-Desalkylquetiapine	4 – 600	y = 0.00996 x—0.0049	0.9972
Risperidone	0.3 – 90	y = 7.1e-5 x^2 + 0.126 x + 0.00203	0.9975
Sulpiride	48 – 2400	y = 0.000198 x—9.94e-5	0.9974
Zotepine	2.5 – 750	y = 0.00105 x + 0.000421	0.9974

Compounds	MF (%)	IS	IS normalized MF (%)
(b) Matrix factor values of all compounds and IS normalized values of analytes			
Aripiprazole	70.8	Desipramine-d_3_	83.5
Dehydroaripiprazole	71.5	Desipramine-d_3_	84.3
Asenapine	110	Amitriptyline-d_3_	115
Blonanserin	170	Blonanserin-d_5_	111
Brexpiprazole	82.8	Quetiapine-d_8_	87.9
Chlorpromazine	98.2	Clomipramine-d_3_	103
Clozapine	88.3	Clozapine-d_8_	98.5
* N*-Desmethylclozapine	92.1	Clozapine-d_8_	103
Clozapine-*N*-oxide	87.6	Desipramine-d_3_	103
Levomepromazine	102	Amitriptyline-d_3_	107
Lurasidone	164	Blonanserin-d_5_	107
Olanzapine	79.3	Olanzapine-d_3_	93.8
Paliperidone	80.5	Olanzapine-d_3_	95.3
Perospirone	109	Clomipramine-d_3_	114
Perphenazine	89.2	Quetiapine-d_8_	94.7
Quetiapine	95.4	Quetiapine-d_8_	101
* N*-Desalkylquetiapine	90.3	Quetiapine-d_8_	95.9
Risperidone	94.2	Clozapine-d_8_	105
Sulpiride	54.1	Olanzapine-d_3_	64.0
Zotepine	130	Blonanserin-d_5_	85.0
Blonanserin-d_5_	153	–	–
Clozapine-d_8_	89.7	–	–
Olanzapine-d_3_	84.5	–	–
Quetiapine-d_8_	94.2	–	–
Amitriptyline-d_3_	95.2	–	–
Clomipramine-d_3_	95.6	–	–
Desipramine-d_3_	84.8	–	–

Analytes	Carryover without wash run (%)	Carryover with wash run (%)
(c) Carryover of analytes without or with wash run		
Aripiprazole	24.4	10.0
Dehydroaripiprazole	27.0	11.3
Asenapine	0	0
Blonanserin	0	0
Brexpiprazole	25.6	6.6
Chlorpromazine	0	0
Clozapine	0	0
* N*-Desmethylclozapine	12.1	4.6
Clozapine-*N*-oxide	0	0
Levomepromazine	0	0
Lurasidone	0	0
Olanzapine	17.3	0
Paliperidone	8.97	5.8
Perospirone	0	0
Perphenazine	0	0
Quetiapine	0	0
* N*-Desalkylquetiapine	24.6	0
Risperidone	10.8	3.9
Sulpiride	0	0
Zotepine	0	0

*MF* matrix factor

### Investigation of analytical conditions in method validation

The analytical method was validated to determine its validity and reliability. It was established that no peaks interfered with the target compound peaks (data not shown). This method exhibits good selectivity. The next step was to investigate the matrix effects [60]. The matrix effect was evaluated for the calculations using MF [61–63]. The results indicated that certain compounds exhibited matrix effects that could be mitigated by IS, except for sulpiride (Table 2b). The calibration curves were then validated. The coefficients of determination (*R*^2^) of the calibration curves were greater than 0.99, indicating a high degree of linearity and suitability for linear and quadratic regressions (Table 2a). Subsequently, the carryover was examined. By administering a series of five injections and a brief run following the analysis of the highest concentration standard sample, it was determined that the peak area of the target compound in the blank sample was less than 20% of the peak area in the lower limit of quantification (LLOQ), indicating that carryover was effectively mitigated (Table 4).

Next, intra- and inter-day reproducibility was verified using QC samples at the LLOQ and three concentrations: the accuracy (RE) for 11 of the 20 compounds (aripiprazole, dehydroaripiprazole, blonanserin, chlorpromazine, clozapine, clozapine-*N*-oxide, levomepromazine, olanzapine, perphenazine, quetiapine, sulpiride, and zotepine) was within ± 15% except for the LLOQ, within ± 20% for the LLOQ, and the precision was less than or equal to 15% for all compounds except for the LLOQ and less than or equal to 20% for the LLOQ. Of the remaining nine compounds, seven were within a maximum of 30% for both accuracy and precision (asenapine, blonanserin, brexpiprazole, *N*-desmethylclozapine, lurasidone, *N*-desalkylquetiapine, and risperidone), and two compounds (paliperidone and perospirone) were within a maximum of ± 25% for accuracy and 40% for precision (Table 3). The stability of the samples was evaluated at three different temperatures: frozen (−80 °C), refrigerated (4 °C), and room temperature (25 °C). The storage temperatures and times varied considerably among the different compounds, with some compounds failing to demonstrate appreciable stability (Table S3). The results indicated that asenapine, blonanserin, chlorpromazine, levomepromazine, and zotepine exhibited a reduction of greater than 50% reduction under at least one condition or concentration (Table S3). The results indicated that brexpiprazole, clozapine, clozapine-*N*-oxide, olanzapine, perospirone, perphezine, risperidone, and sulpiride exhibited a > 20% reduction in concentration under at least one condition or concentration. A possible reason for this reduction in stability is analyte degradation. Degradation is caused by changes in the temperature, light, pH, enzymes, and oxidation. Previous studies have reported that asenapine [36, 37, 64], blonanserin [65], chlorpromazine [66], levomepromazine [67], and zotepine [68] are stable in the plasma. Compounds for which the error exceeded 20%, including brexpiprazole [69], clozapine [10], clozapine-*N*-oxide [10], olanzapine [50, 70], perospirone [71], perphenazine [72], risperidone, [50] and sulpiride [32], were also reported to be stable in plasma. Consequently, it was postulated that the analytes under investigation exhibited sufficient stability in the plasma. The potential cause for this low stability was investigated. We hypothesized that adsorption was a contributing factor. The adsorption of analytes has been shown to result in reduced recovery and poor reproducibility. In contrast to previous studies, our investigation focused on the preparation of samples in tubes. Polypropylene tubes have been used for sampling and storage in numerous published studies. However, numerous studies have employed glass tubes for sample preparation [36, 37, 65, 71]. All storage containers for the stock solutions were made of glass, and there were no differences between the present and previous studies [67, 71, 73]. It can be reasonably inferred from the results that adsorption on polypropylene may be a contributing factor to the observed lower stability (Table S3). The intra- and inter-day reproducibility tests demonstrated that a significant number of compounds exhibited favorable results (Table 2). Although some compounds did not meet the criteria for reproducibility and stability [44], a preliminary test demonstrated that the plasma concentration could be accurately measured using this method.

**Table 3:** Intra- and inter-day reproducibility tests

Analytes	Concentration (ng/mL)	Intra-day		Inter-day	
		RE (%)	CV (%)	RE (%)	CV (%)
Aripiprazole	5	9.43	8.22	8.14	8.82
	7.5	2.56	5.45	4.73	6.29
	75	10.8	1.56	8.58	1.44
	1200	3.89	2.52	2.27	1.78
Dehydroaripiprazole	1	7.92	9.74	7.96	7.15
	1.5	6.33	7.35	5.56	5.14
	15	12.4	2.84	9.07	2.30
	240	2.57	3.23	−1.13	2.67
Asenapine	0.24	1.94	21.1*	−14.5	16.1
	0.48	−2.01	13.0	−7.23	6.22
	1.2	3.19	7.54	−0.139	2.62
	9.6	−8.56	1.77	−7.47	1.68
Blonanserin	0.03	2.89	18.5	−2.11	27.9
	0.06	−5.83	18.9*	−4.81	12.2
	0.15	0.667	8.10	2.33	2.38
	2.4	−6.11	3.10	−2.08	3.09
Brexpiprazole	2	24.2*	3.59	26.9*	4.01
	3	29.1*	5.55	19.1	9.11
	30	10.0	3.24	10.9	2.24
	240	2.78	2.42	−5.37	11.8
Chlorpromazine	5	−3.40	10.4	−0.611	6.67
	7.5	5.53	5.75	2.49	2.82
	75	−3.20	4.75	1.02	3.88
	600	0.222	3.32	2.61	2.11
Clozapine	10	0.783	7.96	−3.51	6.00
	15	−0.778	1.06	5.00	4.63
	150	11.0	2.35	10.2	3.70
	2400	8.96	2.30	4.98	2.85
*N*-Desmethylclozapine	10	24.3	10.9	17.3	4.61
	15	18.8	8.99	18.3*	5.49
	150	9.56	1.60	12.9	2.91
	1200	2.78	3.76	3.24	1.89
Clozapine-*N*-oxide	10	−16.1	6.95	−19.8	4.20
	15	−4.67	5.57	−8.89	3.60
	150	−5.89	3.21	−6.07	2.66
	1200	−12.4	1.50	−7.75	5.59
Levomepromazine	3	−16.3	13.6	−15.1	1.20
	4.5	−5.37	5.10	−11.8	5.16
	45	−9.19	4.68	−6.98	3.73
	720	−7.29	6.09	5.08	8.39
Lurasidone	0.6	23.1	11.5	21.6	4.27
	0.9	19.1*	6.89	27.0*	5.64
	9	18.3*	3.29	15.5*	1.75
	144	12.8	3.98	11.8	2.99
Olanzapine	1.4	5.24	9.50	−1.81	7.35
	2.1	0.238	4.45	6.80	4.48
	21	1.59	3.41	1.77	1.44
	336	−1.64	1.38	0.992	2.33
Paliperidone	0.6	13.2	8.31	18.1	13.4
	0.9	30.4**	9.20	38.7**	14.6
	9	32.8**	2.76	23.4*	5.47
	144	19.9	3.02	16.1	2.61
Perospirone	0.3	−16.6	39.3**	−24.9*	8.91
	0.6	8.17	26.6*	6.76	6.61
	1.5	24.8*	11.3	22.9*	2.13
	12	−6.25	7.41	−6.47	1.87
Perphenazine	0.12	−3.50	12.4	2.49	8.25
	0.24	−1.87	7.32	2.85	3.27
	0.6	−6.78	6.78	−2.14	4.22
	9.6	0.747	2.97	−0.330	1.34
Quetiapine	8	7.38	4.26	5.44	1.71
	12	6.39	3.55	8.94	1.66
	120	−2.78	2.70	0.463	2.28
	1920	6.25	3.06	6.22	0.901
*N*-Desalkylquetiapine	4	19.2	5.50	15.4	3.46
	6	18.7*	4.90	18.6*	1.80
	60	10.2	2.02	13.4	1.98
	480	13.1	2.53	8.33	5.52
Risperidone	0.3	20.6	4.71	20.7	3.57
	0.45	24.6*	3.19	23.3*	3.73
	4.5	17.5*	2.85	19.0*	0.907
	72	11.3	3.66	11.8	4.19
Sulpiride	48	−16.7	18.2	−15.7	14.4
	96	−3.40	10.6	−0.909	2.67
	240	11.3	3.92	6.90	3.73
	1920	6.60	2.91	7.84	4.38
Zotepine	2.5	−0.467	7.62	−2.09	2.74
	3.75	6.40	6.96	5.85	0.367
	37.5	0.933	3.13	1.20	1.70
	600	−8.97	2.32	−2.82	4.69

*CV* coefficient of variation, *RE* relative error. * means its CV or RE is between ± 15% and ± 30%.; ** means that its CV or RE is between ± 30% and ± 40%

### Analysis of antipsychotic drugs in the plasma of patients with schizophrenia

To ascertain the efficacy of this approach, the plasma drug concentrations were analyzed in patients with schizophrenia. Plasma samples were obtained from patients with schizophrenia at Tohoku University Hospital and Shiga Medical University Hospital. Five samples from patients with schizophrenia were analyzed. All the analytes were analyzed within the calibration ranges (Tables 2a, 4). It was proposed that the calibration ranges utilized in this study were suitable for monitoring the plasma concentrations of antipsychotic drugs. The plasma concentrations were outside the therapeutic range, except in patient 5 [19]. The plasma concentration of clozapine is associated with several serious adverse drug reactions, including agranulocytosis, myocarditis, and epileptic seizures [74–77]. All patients receiving clozapine (patient numbers 1–3) had concentrations above the therapeutic range (Table 4). The DDI may be caused by concomitant use of a CYP1A2 inhibitor such as fluvoxamine [22]. Patient 3 was concomitantly administered with brexpiprazole. There is no evidence suggesting that CYP1A2 expression was inhibited. Consequently, it was necessary to conduct a comprehensive analysis of the plasma concentrations of antipsychotics. Patient 4, who was taking 4 mg of risperidone, exhibited higher total concentrations of risperidone and paliperidone (total: 97.9 ng/mL) than the therapeutic range (total: 20–60 ng/mL) [19, 78]. The ratio of paliperidone (9-hydroxyrisperidone) to risperidone is modulated by the enzymatic activity of CYP2D6 [79]. In patient 5, the total concentration of aripiprazole and dehydroaripiprazole was 299.3 ng/mL, which was within the therapeutic range (150–500 ng/mL). The developed method demonstrated the capacity to cover all plasma concentrations within the calibration range (Table 2a). However, in this study, only eight compounds were analyzed in five patients with schizophrenia; the other 12 compounds could not be analyzed. The calibration ranges are expected to be analyzed in real patient plasma samples.

This method is believed to be useful for simultaneous measurement of antipsychotics. However, it is necessary to improve the accuracy of the analytical method, particularly in stability experiments (Table 3b). Nevertheless, this analytical system is useful for the simultaneous measurement of plasma concentrations of antipsychotics approved in Japan.

**Table 4:** Concentrations of antipsychotic drugs and active metabolites in the plasma of schizophrenia patients

Patient number	Dose (mg/day)	Clozapine (ng/mL)	*N*-Desmethylclozapine (ng/mL)	Clozapine-*N*-oxide (ng/mL)	Brexpiprazole (ng/mL)	Aripiprazole (ng/mL)	Dehydroaripiprazole (ng/mL)	Risperidone (ng/mL)	Paliperidone (ng/mL)
1	Clozapine: 400	683	551	99.7					
2	Clozapine: 500	616	342	79.8					
3	Clozapine: 475 Brexpiprazole: 2	1200	655	83.3	111				
4	Risperidone: 6							0.900	97.0
5	Aripiprazole: 18					249	50.3		
Therapeutic range (ng/mL)		350-600	–	–	40-140	100-350	Dehydroaripiprazole+Aripiprazole: 150-500	Risperidone+Paliperidone: 20-60	20-60

## Conclusion

The objective of this study was to verify the usefulness of antipsychotic TDM by elucidating the prescribing trends of antipsychotic drugs at the Tohoku University Hospital and to develop a simultaneous analytical method for antipsychotics approved in Japan and their metabolites. First, a survey was conducted to determine the prevalence of prescription trends. Approximately, one-third of the prescriptions were combination therapies and concomitant drugs that may interact with antipsychotic drugs. Subsequently, the MS/MS conditions were optimized, and the LC conditions were investigated. The analytical method validation tests demonstrated that the method exhibited a certain degree of reliability, although there were issues with reproducibility and storage stability. The method was applied to the measurement of plasma drug concentrations in patients with schizophrenia, and the results demonstrated that the method may be useful for measuring drug plasma concentrations in patients with schizophrenia. The principal novelty of this study was the simultaneous analysis of antipsychotics approved in Japan. Another noteworthy aspect is the adjustment of the calibration curves using CED and IS-CID. The application of this method will permit the verification of the relationship between blood concentrations of antipsychotics and their side effects. It is anticipated that this will contribute to the optimization of schizophrenia treatment.

### Supplementary Information

Below is the link to the electronic supplementary material.

Supplementary file1 (DOCX 29 KB)

Supplementary file2 (DOCX 28 KB)

Supplementary file3 (DOCX 31 KB)

## Data Availability

For details other than the published data including the Supplmentary items, please contact the authors.

## Abbreviations

CAD: Collision gas
CE: Collision energy
CV: Coefficient of variation
CXP: Cell exit potential
DDI: Drug–drug interaction
DP: Declustering potential
EP: Entrance potential
ESI: Electrospray ionization
HPLC: High-performance liquid chromatography
IS: Internal standard
LC: Liquid chromatography
LC/MS/MS: Liquid chromatography/tandem mass spectrometry
MS: Mass spectrometry
MS/MS: Tandem mass spectrometry
QC: Quality control
SRM: Selected reaction monitoring
