# Supplementary material for: Development of a simultaneous LC–MS/MS analytical method for plasma: 16 antipsychotics approved in Japan and 4 drug metabolites
Source: Anal Sci. 2024 Jun 25;40(9):1749–63. doi: 10.1007/s44211-024-00619-2 (PMC11358186; doi:10.1007/s44211-024-00619-2)
Supplement: Supplementary file 1 — Supplementary file1 (DOCX 29 KB) [file 44211_2024_619_MOESM1_ESM.docx]

Table S1 The analyte concentrations of all compounds

1. Calibration curves

| No | Compounds | Lv1  (ng/mL) | Lv2  (ng/mL) | Lv3  (ng/mL) | Lv4  (ng/mL) | Lv5  (ng/mL) | Lv6  (ng/mL) | Lv7  (ng/mL) | Lv8  (ng/mL) |
| --- | --- | --- | --- | --- | --- | --- | --- | --- | --- |
| 1 | Aripiprazole | 5 | 15 | 25 | 50 | 150 | 250 | 500 | 750 |
| 2 | Dehydroaripiprazole | 1 | 3 | 5 | 10 | 30 | 50 | 100 | 150 |
| 3 | Asenapine | 0.08 | 0.24 | 0.4 | 0.8 | 2.4 | 4 | 8 | 12 |
| 4 | Blonanserin | 0.01 | 0.03 | 0.05 | 0.1 | 0.3 | 0.5 | 1 | 1.5 |
| 5 | Brexpiprazole | 2 | 6 | 10 | 20 | 60 | 100 | 200 | 300 |
| 6 | Chlorpromazine | 5 | 15 | 25 | 50 | 150 | 250 | 500 | 750 |
| 7 | Clozapine | 10 | 30 | 50 | 100 | 300 | 500 | 1000 | 1500 |
| 8 | *N*-Desmethylclozapine | 10 | 30 | 50 | 100 | 300 | 500 | 1000 | 1500 |
| 9 | Clozapine-*N*-oxide | 10 | 30 | 50 | 100 | 300 | 500 | 1000 | 1500 |
| 10 | Levomepromazine | 3 | 9 | 15 | 30 | 90 | 150 | 300 | 450 |
| 11 | Lurasidone | 0.6 | 1.8 | 3 | 6 | 18 | 30 | 60 | 90 |
| 12 | Olanzapine | 1.4 | 4.2 | 7 | 14 | 42 | 70 | 140 | 210 |
| 13 | Paliperidone | 0.6 | 1.8 | 3 | 6 | 18 | 30 | 60 | 90 |
| 14 | Perospirone | 0.1 | 0.3 | 0.5 | 1 | 3 | 5 | 10 | 15 |
| 15 | Perphenazine | 0.04 | 0.12 | 0.2 | 0.4 | 1.2 | 2 | 4 | 6 |
| 16 | Quetiapine | 8 | 24 | 40 | 80 | 240 | 400 | 800 | 1200 |
| 17 | *N*-Desalkylquetiapine | 4 | 12 | 20 | 40 | 120 | 200 | 400 | 600 |
| 18 | Risperidone | 0.3 | 0.9 | 1.5 | 3 | 9 | 15 | 30 | 45 |
| 19 | Sulpiride | 16 | 48 | 80 | 160 | 480 | 800 | 1600 | 2400 |
| 20 | Zotepine | 2.5 | 7.5 | 12.5 | 25 | 75 | 125 | 250 | 375 |

1. Quality control samples

| No | Compounds | LLQC  (ng/mL) | LQC  (ng/mL) | MQC  (ng/mL) | HQC  (ng/mL) |
| --- | --- | --- | --- | --- | --- |
| 1 | Aripiprazole | 5 | 7.5 | 75 | 1200 |
| 2 | Dehydroaripiprazole | 0.4 | 1.5 | 15 | 240 |
| 3 | Asenapine | 0.24 | 0.48 | 1.2 | 9.6 |
| 4 | Blonanserin | 0.03 | 0.06 | 0.15 | 2.4 |
| 5 | Brexpiprazole | 2 | 3 | 30 | 240 |
| 6 | Chlorpromazine | 5 | 7.5 | 75 | 600 |
| 7 | Clozapine | 10 | 15 | 150 | 2400 |
| 8 | *N*-Desmethylclozapine | 10 | 15 | 150 | 1200 |
| 9 | Clozapine-*N*-oxide | 10 | 15 | 150 | 1200 |
| 10 | Levomepromazine | 3 | 4.5 | 45 | 720 |
| 11 | Lurasidone | 0.6 | 0.9 | 9 | 144 |
| 12 | Olanzapine | 1.4 | 2.1 | 21 | 336 |
| 13 | Paliperidone | 0.6 | 0.9 | 9 | 144 |
| 14 | Perospirone | 0.3 | 0.60 | 1.5 | 12 |
| 15 | Perphenazine | 0.12 | 0.24 | 0.6 | 9.6 |
| 16 | Quetiapine | 8 | 12 | 120 | 1920 |
| 17 | *N*-Desalkylquetiapine | 4 | 6 | 60 | 480 |
| 18 | Risperidone | 0.3 | 0.45 | 4.5 | 72 |
| 19 | Sulpiride | 48 | 96 | 240 | 1920 |
| 20 | Zotepine | 2.5 | 3.75 | 37.5 | 600 |

LLQC, lowest quality control; LQC, low quality control; MQC, middle quality control; HQC, high quality control.
