# Supplementary material for: Development of a simultaneous LC–MS/MS analytical method for plasma: 16 antipsychotics approved in Japan and 4 drug metabolites
Source: Anal Sci. 2024 Jun 25;40(9):1749–63. doi: 10.1007/s44211-024-00619-2 (PMC11358186; doi:10.1007/s44211-024-00619-2)
Supplement: Supplementary file 2 — Supplementary file2 (DOCX 28 KB) [file 44211_2024_619_MOESM2_ESM.docx]

Table S2 Analysis result of prescription of Tohoku University Hospital

1. Prescription number of antipsychotic drugs

| Drug | Number  (n) | The number of concomitant drugs  (n) |
| --- | --- | --- |
| Aripiprazole | 325 | 393 |
| Risperidone | 292 |  |
| Quetiapine | 259 | 454 |
| Olanzapine | 211 | 348 |
| Levomepromazine | 194 |  |
| Brexpiprazole | 91 |  |
| Chlorpromazine | 80 |  |
| Paliperidone | 63 |  |
| Sulpiride | 62 |  |
| Blonanserin | 57 |  |
| Zotepine | 29 |  |
| Asenapine | 26 | 123 |
| Perospirone | 24 |  |
| Lurasidone | 19 |  |
| Clozapine | 13 | 65 |
| Perphenazine | 6 |  |
| All | 1751 |  |

1. Concomitant drug prescriptions with potential drug-drug interaction via cytochrome P450

|  | Asenapine  (n) | Aripiprazole  (n) | Olanzapine  (n) | Quetiapine  (n) | Chlorpromazine  (n) | zotepine  (n) | Pariperidone  (n) | Brexpiprazole  (n) | Blonanserin  (n) | Perospirone  (n) | Risperidone  (n) | Lurasidone  (n) | Levomepromazine  (n) | Total  (n) |
| --- | --- | --- | --- | --- | --- | --- | --- | --- | --- | --- | --- | --- | --- | --- |
| Asenapine |  | 2 |  |  | 1 |  | 2 | 7 |  |  | 5 |  | 3 | 20 |
| Amiodarone |  |  |  | 1 |  |  |  |  |  |  | 1 |  |  | 2 |
| Omeprazole | 1 |  | 2 |  |  |  |  |  |  |  |  |  |  | 3 |
| Carbamazepine | 1 | 9 | 13 | 11 |  |  | 1 | 3 |  |  |  |  |  | 38 |
| Clarithromycin |  | 2 |  |  |  | 1 |  |  |  |  |  | 1 |  | 4 |
| Clobazam |  |  |  | 6 |  |  | 1 |  |  |  |  |  |  | 7 |
| Cyclosporine |  |  |  | 4 |  |  |  |  | 3 |  |  |  |  | 7 |
| Diltiazem |  | 1 |  |  |  |  |  |  |  |  |  |  |  | 1 |
| Sertraline |  | 28 |  |  | 6 |  | 2 | 9 |  |  | 10 |  | 14 | 69 |
| Dexamethasone |  | 2 |  | 4 |  | 1 |  |  |  |  |  | 2 |  | 9 |
| Terbinafine |  | 2 |  |  |  |  | 1 | 2 |  |  | 4 |  | 1 | 10 |
| Paroxetine |  | 8 |  |  | 5 |  |  | 2 |  |  | 5 |  | 5 | 25 |
| Haloperidol |  | 4 |  |  | 3 |  | 3 | 3 |  |  | 5 |  | 35 | 53 |
| Phenytoin |  | 2 |  |  |  |  | 2 |  |  |  |  |  |  | 4 |
| Phenobarbital |  |  |  |  |  |  | 1 |  |  |  |  |  |  | 1 |
| Fluconazole |  |  |  |  |  |  |  |  | 1 |  |  |  |  | 1 |
| Fluvoxamine | 1 | 15 | 7 | 22 | 4 |  |  | 2 | 1 | 1 | 8 |  | 5 | 66 |
| Bromocriptine |  |  |  | 2 |  |  | 2 |  |  |  |  |  |  | 4 |
| Verapamil |  |  |  | 1 |  |  |  |  |  |  |  |  |  | 1 |
| Voriconazole |  |  |  | 1 |  |  |  |  |  |  |  |  |  | 1 |
| Modafinil |  | 1 |  |  |  |  |  |  |  |  |  |  |  | 1 |
| Levomepromazine |  | 32 |  |  | 4 |  | 9 | 12 |  |  | 46 |  |  | 103 |
| Total | 3 | 108 | 22 | 52 | 23 | 2 | 24 | 40 | 5 | 1 | 84 | 3 | 63 | 430 |
