# Supplementary material for: Development of a simultaneous LC–MS/MS analytical method for plasma: 16 antipsychotics approved in Japan and 4 drug metabolites
Source: Anal Sci. 2024 Jun 25;40(9):1749–63. doi: 10.1007/s44211-024-00619-2 (PMC11358186; doi:10.1007/s44211-024-00619-2)
Supplement: Supplementary file 3 — Supplementary file3 (DOCX 31 KB) [file 44211_2024_619_MOESM3_ESM.docx]

Table S3 Stability tests of analytes in plasma

| Analytes | Conc.  (ng/mL) | -80℃ | | 4℃ | | 25℃ | |
| --- | --- | --- | --- | --- | --- | --- | --- |
|  |  | Dur.  (day) | RE  (%) | Dur.  (h) | RE  (%) | Dur.  (h) | RE  (%) |
| Aripiprazole | 7.5 | 28 | -8.04 | 24 | -0.444 | 2 | -4.22 |
|  | 1200 |  | 2.78 |  | 7.78 |  | -20.3 |
| Dehydroaripiprazole | 1.5 | 28 | -12.7 | 24 | -4.44 | 2 | 1.11 |
|  | 240 |  | -2.92 |  | -3.75 |  | -27.5* |
| Asenapine | 0.48 | 1 | -58.1*** | 2 | -50.9*** | 2 | -52.4*** |
|  | 9.6 |  | -24.2 |  | -26.5 |  | -23.5 |
| Blonanserin | 0.06 | 1 | -41.5** | 2 | -48.7** | 2 | -20.8 |
|  | 2.4 |  | -4.03 |  | -12.4 |  | -6.53 |
| Brexpiprazole | 3 | 7 | 3.33 | 2 | 7.56 | 2 | 17.1 |
|  | 240 |  | -12.9 |  | -15.6 |  | -24.7 |
| Chlorpromazine | 7.5 | 1 | -43.6 | 2 | -57.5 | 2 | -36.6 |
|  | 600 |  | -17.1 |  | -26.9 |  | -30.3 |
| Clozapine | 15 | 1 | -21.3 | 2 | -20.0 | 24 | -6.22 |
|  | 2400 |  | -0.417 |  | -2.92 |  | -4.44 |
| *N*-Desmethyl clozapine | 15 | 7 | -0.444 | 6 | -14.0 | 2 | 24.7 |
|  | 1200 |  | -7.50 |  | -2.22 |  | -18.5 |
| Clozapine-*N*-oxide | 15 | 1 | -29.3 | 2 | -20.9 | 2 | -23.3 |
|  | 1200 |  | -9.72 |  | -4.72 |  | -28.9 |
| Levomepromazine | 4.5 | 1 | -51.8 | 2 | -62.2 | 2 | -50.1 |
|  | 720 |  | 2.96 |  | 3.15 |  | -9.49 |
| Lurasidone | 0.9 | 1 | -8.00 | 2 | -13.9 | 24 | 9.48 |
|  | 144 |  | -0.463 |  | 5.32 |  | 8.10 |
| Olanzapine | 2.1 | 1 | -28.3 | 2 | -18.6 | 2 | -10.6 |
|  | 336 |  | -1.09 |  | -0.694 |  | -0.893 |
| Paliperidone | 0.9 | 1 | 12.0 | 24 | -3.96 | 2 | 17.1 |
|  | 144 |  | 19.9 |  | -6.71 |  | 18.5 |
| Perospirone | 0.6 | 1 | -7.94 | 2 | -6.22 | 2 | -12.9 |
|  | 12 |  | -15.4 |  | -24.2 |  | -30.6 |
| Perphenazine | 0.24 | 1 | -19.6 | 2 | -20.3 | 24 | -10.6 |
|  | 9.6 |  | 1.25 |  | 12.5 |  | 3.82 |
| Quetiapine | 12 | 1 | -5.83 | 2 | -11.6 | 24 | -2.50 |
|  | 1920 |  | 4.17 |  | 7.12 |  | 8.85 |
| *N*-Desalkylquetiapine | 6 | 14 | -2.44 | 2 | -18.7 | 24 | -5.89 |
|  | 480 |  | 6.67 |  | -5.63 |  | -5.69 |
| Risperidone | 0.45 | 28 | -3.70 | 2 | -3.78 | 2 | 18.4 |
|  | 72 |  | 10.6 |  | 23.6 |  | 26.7 |
| Sulpiride | 96 | 1 | 25.3 | 24 | -10.0 | 2 | -19.3 |
|  | 1920 |  | 7.64 |  | -14.2 |  | -19.6 |
| Zotepine | 3.75 | 1 | -44.1 | 2 | -60.6 | 2 | -40.4 |
|  | 600 |  | -12.9 |  | -12.2 |  | -18.2 |

Conc., concentration; Dur., duration. * means its CV or RE between ±15% and ±30%.; ** means that its CV or RE are between ±30% and ±40%, and *** means that its CV or RE are more than ±40%, respectively.
